# Orthodontics in Growing Patients: Clinical/Biological Evidence and Technological Advancement 2018

**DOI:** 10.1155/2018/7281846

**Published:** 2018-11-28

**Authors:** Simona Tecco, Alberto Baldini, Enita Nakaš, Jasmina Primozic

**Affiliations:** ^1^Dental School, Vita-Salute University and Department of Dentistry, IRCCS San Raffaele Hospital, Milan, Italy; ^2^University of Tor Vergata, Rome, Italy; ^3^University of Sarajevo, Sarajevo, Bosnia and Herzegovina; ^4^University Clinical Centre of Ljubljana, Ljubljana, Slovenia

Interceptive orthodontics (the so-called early orthodontic treatment) is common in the world [1]. With the recent improvement in diagnostic tools, novel potential therapeutic devices and techniques have been recognized; it became a well-established strategy and found several applications in clinics or under clinical trials [2, 3]. However, the most significant barrier to providing interceptive orthodontic care by dental practitioners is still the lack of self-confidence relating to the effectiveness of their chosen treatment plan [1, 2]. There remains a lot to be learned about the effective therapeutic results, and so, numerous forms of therapeutic approaches interceptive orthodontics are still explored today [1, 2, 4, 5].

As the interceptive orthodontic with the right timing has demonstrated success in the treatment of some types of malocclusion [3, 6], the study of the timing has been considered a promising new approach to therapy for many years [7, 8]. Recently, new insights into the technique to decide the timing of an interceptive orthodontic have been explored [9, 10]. Besides, novel biological effects of already-known therapeutic devices and/or techniques have attracted much more attention in the recent years [11–13]. The occlusion of teeth is involved in regulation of some functional receptors by costimulatory or inhibitory signaling transduction in the other apparatus (for example, the visual apparatus) and the knowledge of these correlations has achieved great success among clinicians in the last years [14].

However, it is not yet clear, from the results of the literature, whether early treatment is desirable because tissue tolerance and its power of adjustment are at or near their maximum, or because there is no assurance that early treatment will be helpful, without causing an unnecessary lengthening of the time of treatment. Also, from the emerging trends in orthodontics and dentofacial orthopedics, early treatment not only prolongs therapy but also may exhaust the child's spirit of cooperation and compliance.

Many researchers believe that functional appliances can influence mandibular and maxillary growth. Some histological evidence supports this concept, and ample clinical evidence has been produced in attempts to show that the use of functional appliances can alter the skeletal relationship of the jaws [12]. However, this evidence does not always take into account the effects of the normal growth of TMJ [15].

This special issue encompasses cutting-edge research and review articles focusing on the role of the potential new therapeutic devices and technique for the treatment of malocclusion in growing patients. It includes articles describing the advance of interceptive orthodontics, summarized as follows.

**Four studies focus on the craniofacial anatomy and its relationship with the interceptive orthodontics, for both diagnosis and therapeutically approaches.**

*Dr. P. M. Ortiz *et al. investigate the association between unilateral/bilateral maxillary canine impaction and sella-turcica bridging using CBCT imaging, analyzing 76 CBCT images of the craniofacial complex including sella-turcica. Although the odds for unilateral canine impaction that resulted increased in the right and left sella-turcica bridging groups, compared to the controls, the difference was not statistically significant. Therefore, in contrast with previous 2D studies, these authors finally clarify through a 3D visual approach that there is no statistically significant association between unilateral/bilateral palatal canine impaction and sella-turcica bridging.

*Dr. A. Przystańska *et al. evaluate CT images to assess the age-related changes in maxillary sinus diameters with the diameters of the facial skeleton, in a sample of 170 patients aged 0–18 years (85 females and 85 males). The maxillary sinuses of every patient were bilaterally measured in three planes, and the conclusion was that, in females, the correlation between the sinus diameter and the facial skeleton was very strong and related to the age of the subject and to the female gender. Therefore, the authors finally clarify that all measurements of maxillary sinuses correlate with midface dimensions.

These two studies once again highlight the importance of 3D visualization in interceptive orthodontics, already known in the literature [15, 16].

*Dr. A. Baldini *et al. reviewed the published clinical data about the already known correlation between the visual apparatus and the dental occlusion. However, with respect to the previous literature, these authors finally clarify that there is only a* middle level* of evidence of such type of correlation. That concern disturbs such as ocular disorders (myopia, hyperopia, astigmatism, exophoria, and an unphysiological gait due to ocular convergence defects) and dental occlusion in class II, although it has not been possible to establish a cause-effect relationship yet.

On the same field,* Dr. S. Caruso *et al. report the existence of correlations between dysfunctions related to visual impairments and dental occlusion in a sample of 34 children (21 males and 13 females; mean age 11 ± 2 years) that underwent visual clinical tests to evaluate the presence of fusional vergence defects and amplitude. Finally, the authors describe a statistically significant association between the molar occlusal relationship and the occurrence of exodeviations.

**Three studies concern the technical procedures during interceptive orthodontic treatment.**

*Dr. G. Perinetti *et al. report a study on the indices of sagittal jaw relationship (ANB angle, *β* angle, and MMBP-Wits) that are, as already known, all subjected to a geometrical distortion, especially from facial divergence, making the use of floating (individualized) norms necessary. So the authors provide useful floating norms for the ANB angle and—for the first time in literature—for the *β* angle and MMBP-Wits. In addition, the authors also clarify that while the ANB angle is subjected to significantly more geometrical distortion as compared to the *β* angle and MMBP-Wits, floating norms may be used to individualize the reference values for both the *β* angle and MMBP-Wits.

*Dr. M. Çifter* investigates one of the most common procedures in interceptive orthodontics, that is, the dental photography procedure. He reports the point of view of patients on the base of their experience with this procedure. The author concludes that the lack of detailed information regarding this procedure and some instruments (intraoral mirrors and retractors) could represent the primary causes of patient stress before the procedure. So, he suggests that patients must be informed in advance and in detail about this (*only apparently harmless*) procedure for the patient and the equipment to be used, to maximize both patient satisfaction and image quality.

*Dr. S. Mummolo *et al. have dealt with a particular category of patients who are increasingly subjected to interceptive orthodontics, which are the oral breathers, usually treated with palatal expansion [7, 11]. The authors present an observational 6-month case-control study aimed to estimate plaque index, salivary flow, buffering capacity of saliva, and specific Streptococcus mutants and Lactobacillus rates in a mouth breathing late adolescents sample. They found that the mouth breathing subjects show a significantly higher risk to develop Streptococcus mutants bacterial loading with respect to nasal breathing subjects. Thus, these data give strength to the actual clinical guidelines that recommend an early interceptive orthodontic treatment in growing subjects with mouth breathing, through a palatal expansion, to improve their nasal air flow.

Two studies report interesting data on the therapeutic procedure for class III and class II malocclusion treated with interceptive orthodontics.

*Dr. R. Clemente *et al. present a review that addresses the comparative effects of skeletal anchored maxillary protraction versus dental anchored in the orthodontic treatment of class III patients. On the base of their review, the authors finally clarify a famous controversy about the anchorage of the forces in interceptive orthodontics. They state that a higher proclination of the upper incisors in the group treated with a dental anchorage facial mask, as compared to that treated with skeletal anchorage.

Also, almost all the studies indicated a greater maxillary advancement in the group treated with skeletal anchorage.

*Dr. F. Gazzani *et al. present a study on the correction of class II malocclusion. They assess the three-dimensional (3D) maxillomandibular and dental response to Balters Bionator (BB) and the Sander Bite Jumping Appliance (SBJA) in growing patients, evaluating twenty-seven class II division 1 patients (13 males, 14 females), consecutively treated with either the BB or SBJA.

Patients treated with the SBJA and BB orthopedic appliances presented, respectively, 4.7 mm and 4.5 mm of 3D displacement of the chin, with marked ramus growth of, respectively, 3.7 mm and 2.3 mm. While the mandible and maxilla grew downward and forward, no opening of the mandible plane was observed. Both appliances adequately controlled the labial inclination of lower incisors (1.3° and 0.3°, for the SBJA and BB groups, resp.). In conclusion, the authors state that the maxillomandibular and dental growth responses to BB and SBJA therapies are characterized by vertical ramus growth and elongation of the mandible that improves the maxillomandibular relationship with adequate control of lower incisor position.

Finally, about the new appliances, Dr. N. Ozkalayci and Dr. M. Yetmez present a new adjustable Cise space maintainer for preventive orthodontic applications. It is a stainless steel based new design which consists of various components. The authors describe why the space maintainer is stable and is used for maintaining and/or regaining the space which arouses early loss of the molar tooth.
